# Dietary Zinc Differentially Regulates the Effects of the GPR39 Receptor Agonist, TC-G 1008, in the Maximal Electroshock Seizure Test and Pentylenetetrazole-Kindling Model of Epilepsy

**DOI:** 10.3390/cells12020264

**Published:** 2023-01-09

**Authors:** Urszula Doboszewska, Katarzyna Socała, Mateusz Pieróg, Dorota Nieoczym, Jan Sawicki, Adam Sajnóg, Bernadeta Szewczyk, Katarzyna Mlyniec, Ireneusz Sowa, Danuta Barałkiewicz, Piotr Wlaź

**Affiliations:** 1 Department of Animal Physiology and Pharmacology, Institute of Biological Sciences, Maria Curie-Skłodowska University, Akademicka 19, PL 20-033 Lublin, Poland; 2 Department of Pharmacobiology, Jagiellonian University Medical College, Medyczna 9, PL 30-688 Kraków, Poland; 3 Department of Analytical Chemistry, Medical University of Lublin, Chodźki 4A, PL 20-093 Lublin, Poland; 4 Department of Trace Analysis, Adam Mickiewicz University, Uniwersytetu Poznanskiego 8, PL 61-614 Poznan, Poland; 5 Department of Neurobiology, Maj Institute of Pharmacology, Polish Academy of Sciences, Smetna 12, PL 31-343 Krakow, Poland

**Keywords:** zinc chloride, valproic acid, serum zinc, hippocampus, CREB, BDNF, TrkB

## Abstract

The G-protein coupled receptor 39 (GPR39) is gaining increasing attention as a target for future drugs, yet there are gaps in the understanding of its pharmacology. Zinc is an endogenous agonist or an allosteric modulator, while TC-G 1008 is a synthetic, small molecule agonist. Zinc is also a positive allosteric modulator for the activity of TC-G 1008 at GPR39. Activation of GPR39 by TC-G 1008 facilitated the development of epileptogenesis in the pentylenetetrazole (PTZ)-induced kindling model of epilepsy. Congruently, TC-G 1008 decreased the seizure threshold in the maximal electroshock seizure threshold (MEST) test. Here, we investigated the effects of TC-G 1008 under the condition of zinc deficiency. Mice were fed a zinc-adequate diet (ZnA, 50 mg Zn/kg) or a zinc-deficient diet (ZnD, 3 mg Zn/kg) for 4 weeks. Following 4 weeks of dietary zinc restriction, TC-G 1008 was administered as a single dose and the MEST test was performed. Additional groups of mice began the PTZ-kindling model during which TC-G 1008 was administered repeatedly and the diet was continued. TC-G 1008 administered acutely decreased the seizure threshold in the MEST test in mice fed the ZnD diet but not in mice fed the ZnA diet. TC-G 1008 administered chronically increased the maximal seizure severity and the percentage of fully kindled mice in those fed the ZnA diet, but not in mice fed the ZnD diet. Our data showed that the amount of zinc in a diet is a factor contributing to the effects of TC-G 1008 in vivo.

## 1. Introduction

G-protein coupled receptors (GPCRs) constitute the largest group of membrane proteins targeted by the approved drugs, but medications still work only on a small number of receptors belonging to this superfamily [1,2,3]. Orphan receptors represent potential new targets for pharmacological interventions [4]. G-protein coupled receptor 39 (GPR39) belongs to orphan GPCRs of class A [5]. Several endogenous ligands have been proposed [6,7,8,9] including the essential element zinc as agonist [10,11]. Yet, there is insufficient understanding of the receptor pharmacology and studies have only just begun to unravel its relevance in physiology and pathophysiology.

The role of GPR39 in acute seizures was suggested in a few animal studies. Lithium chloride-pilocarpine-induced status epilepticus (SE) decreased the expression of GPR39 at the protein level in the hippocampus [12]. RNA sequencing showed up-regulation of the *gpr39* gene in *stim2b* knockout zebrafish, which is hyperactive and more sensitive to treatment with pentylenetetrazole (PTZ) [13]. GPR39 knockout (KO) mice (bred on C57BL/6 genetic background) exhibited enhanced susceptibility to acute seizures induced by a single intraperitoneal (i.p.) injection of kainic acid (KA), compared with wild-type (WT) littermates [14,15]. However, we found that GPR39 KO mice (bred in mixed genetic background C57BL/6 × CBA) did not differ from WT mice in terms of the seizure threshold in the maximal electroshock seizure threshold (MEST) test or the maximal seizure severity in the PTZ-induced kindling model of epilepsy [16]. Thus, the genotype had no effect on either acute electrically induced seizures or on the chronic process of epilepsy development, i.e., epileptogenesis [17], induced by a chemoconvulsant, PTZ [16]. 

Evidence for the role of GPR39 in epileptogenesis follows the employment of a ligand for GPR39. TC-G 1008 (compound 3 [18], GPR39-C3 [19]) is the most widely used, synthetic, small molecule agonist [20]. It acts via G_s_, G_q,_ G_12/13_, and β-arrestin pathways [18,19]. We showed that the concentrations of TC-G 1008 attained in the brain tissue after i.p. administration in mice were sufficient to occupy the receptor. Thus, the behavioral effects of TC-G 1008 may be attributed to action at GPR39. Furthermore, by combining the observation on the chronic effects on behavioral seizures in genetically unmodified mice and GPR39 KO mice (C57BL/6 × CBA background) we demonstrated that TC-G 1008 facilitated PTZ-induced epileptogenesis via GPR39. Hence, the genotype per se did not affect epileptogenesis but the pharmacological activation of GPR39 aggravated epileptogenesis in the PTZ-kindling model. The effects of TC-G 1008 on PTZ-epileptogenesis were mediated selectively by GPR39, but we concomitantly observed the non-selective activity of TC-G 1008 upon GPR39 on cyclic-AMP-response element binding protein (CREB) activation in the hippocampus. In genetically unmodified mice TC-G 1008 decreased the seizure threshold in the MEST test [16]. 

Zinc is an allosteric modulator at numerous receptors [21]. The neuromodulatory function is the best documented among zinc signaling properties. The bio-metal has been suggested as an endogenous agonist but it is plausible that it is a positive allosteric modulator (PAM) for the activity of another endogenous agonist at GPR39. In addition, zinc is a PAM for the activity of TC-G 1008 at GPR39 [19]. Zinc is a PAM also for other agonists of GPR39, namely kinase inhibitors: LY2784544 and GS2636771 [19], compound TM-N1324 [22], compounds: AZ7914, AZ4237, AZ1395 [23]. All of them are more potent in the presence of zinc. This element is a nutritionally essential metal which is supplied by diet [24]. Zinc-deficient (ZnD) diet decreased the expression of GPR39 at the protein level in the hippocampus [12,25] and frontal cortex [25]. We conjectured that decreased zinc supply may change the effects of GPR39 agonist(s). We therefore examined the impact of TC-G 1008 on the seizure threshold and epileptogenesis under the condition of zinc deficiency which was induced by a ZnD diet as well as on CREB activation in the hippocampus.

## 2. Materials and Methods

### 2.1. Animals and Diet

Housing and experimental procedures were conducted in accordance with the European Union Directive of 22 September 2010 (2010/63/EU), and Polish legislation acts concerning animal experimentation. The protocols were approved by the Local Ethics Committee in Lublin (approval numbers: 38/2017, 48/2018). 

Experimentally naïve male Swiss Albino mice (*n* = 636) with a body weight range of 17–30 g, were purchased from a licensed breeder (Laboratory Animals Breeding, Ilkowice, Poland) and were housed in an animal house at the Faculty of Biology and Biotechnology of Maria Curie-Skłodowska University in Lublin, in groups of 7–8 in open Makrolon cages (37 × 21 × 14 cm) under strictly controlled laboratory conditions (temperature maintained at 21–24 °C, relative humidity at 45–65%) with an artificial 12/12 h light/dark regimen (light on at 6:00 a.m.). The environment was enriched with nest material and paper tubes. 

The animals were randomly assigned to the experimental groups. They were habituated to the laboratory conditions for 1 week prior to use. During the habituation phase, the mice were fed a standard rodent chow diet (Murigran, Agropol S.J., Motycz, Poland) that contained 25 mg Zn/kg. Following the habituation phase, the animals were fed with a zinc-adequate diet (ZnA) of 50 mg Zn/kg or a ZnD diet of 3 mg Zn/kg (Altromin GmbH, Lage, Germany). Diet and tap water were provided ad libitium.

The behavioral procedures began after 4 weeks of the ZnA or ZnD diet administration. They were performed between 8:00 a.m. and 3:00 p.m., after a minimum 30-min adaptation period to the conditions in the experimental room. Blinding was not feasible during behavioral experiments due to the rotations of experimenters who either administered compounds or observed their behavioral effects. Only male mice were used to exclude the possible impact of the estrous cycle on seizure susceptibility [26]. Except 1% (*w*/*v*) ophtalmic solution of tetracaine, which was used for short-term topical ophthalmic anesthesia, no anesthetics or analgesics were used, to reduce the possibility of a pharmacodynamic or pharmacokinetic interaction between these agents and the examined compounds. Each animal was used only once in the acute seizure test/model. During the experiments, the animals were closely followed-up by the animal caretakers and researchers, with regular inspection by a veterinarian, according to the standard health and animal welfare procedures of the local animal facility. All efforts were made to minimize animal suffering as well as the number of animals used in the study. 

### 2.2. Drugs

GPR39 agonists: TC-G 1008 and zinc chloride, ZnCl_2_, were used in the study. Small molecule agonist, TC-G 1008, (*N*-[3-Chloro-4-[[[2-(methylamino)-6-(2-pyridinyl)-4-pyrimidinyl]amino]methyl]phenyl]methanesulfonamide) was purchased from Adooq Bioscience LLC (Irvine, CA, USA). Standard anti-seizure drug, valproic acid (VPA) was utilized as a positive control. VPA (sodium salt), ZnCl_2_ and PTZ were obtained from Sigma-Aldrich. ZnCl_2_, VPA and PTZ were dissolved in physiological saline (0.9% (*w*/*v*) sodium chloride (NaCl) solution). TC-G 1008 was suspended in 1% (*w*/*v*) Tween 80 solution in physiological saline. Drug solutions/suspensions were prepared freshly and administered i.p. at a volume of 0.1 mL per 10 g of body weight. Control groups received vehicle (VEH) (1% Tween 80 in physiological saline). The drugs were administered 30 min before the maximal electroshock seizure (MES)/MEST test or before PTZ injection. This pretreatment time was chosen based on our previous study in which we determined TC-G 1008 concentrations in the serum and brain after i.p. administration of this compound in Swiss Albino mice [16].

### 2.3. Maximal Electroshock Seizures 

MES is a widely used rodent model of acute seizures that produces generalized tonic–clonic seizures [26]. Ophthalmic solution of tetracaine (1%) was administered for short-term topical ophthalmic anesthesia. Then, constant current stimuli (sine-wave pulses at 50 Hz for 200 ms) were applied via saline-soaked transcorneal electrodes with the usage of a rodent shocker (type 221; Hugo Sachs Elektronik, Freiburg, Germany). During stimulation, mice were restrained manually and immediately following stimulation they were placed in a transparent box without bedding for behavioral observation on the presence or absence of seizure activity. Tonic hindlimb extension, defined as the rigid extension of the hindlimb that exceeds a 90° angle with the body, was considered as an endpoint. Two experimental approaches were used: (1) the MEST test that employed stimulation at varied current intensities (7.6–17.4 mA) and (2) MES that employed stimulation at a fixed current intensity (50 mA).

(1). The mice were injected with a single dose of TC-G 1008, ZnCl_2_, VPA or VEH. 30 min later the MEST test was performed. The threshold current was established according to an “up-and-down” method described by Kimball et al. [27]. Current intensity was lowered or raised by 0.06-log intervals depending on whether the previously stimulated animal did or did not exert tonic hindlimb extension, respectively. The data obtained in groups of 20 animals were used to determine the threshold current causing endpoint in 50% of mice (CS_50_ with confidence limits for 95% probability). In the MEST test, the dose–response relationship was assessed. An initial dose of TC-G 1008 or ZnCl_2_ was selected, and the dose was either increased or decreased in a subsequent group of mice, depending on whether the previous dose affected the seizure threshold. The dose of VPA has been established to increase the seizure threshold in this test [28]. Following MEST, the mice were euthanized with >70% carbon dioxide (CO_2_).
(2). Groups of mice (*n* = 10) were injected with a single dose of TC-G 1008, ZnCl_2_, VPA, or VEH. 30 min later they were stimulated with supramaximal MES stimulus of 50 mA. The doses of drugs applied before MES were selected based on the results of the MEST test and our previous study [16]—the effective doses of TC-G 1008 (20 mg/kg) and ZnCl_2_ (16 mg Zn/kg) were administered. Control, non-stimulated (sham) animals received VEH but did not receive MES stimulus.

### 2.4. PTZ Kindling Model of Epilepsy

**Figure 1:**
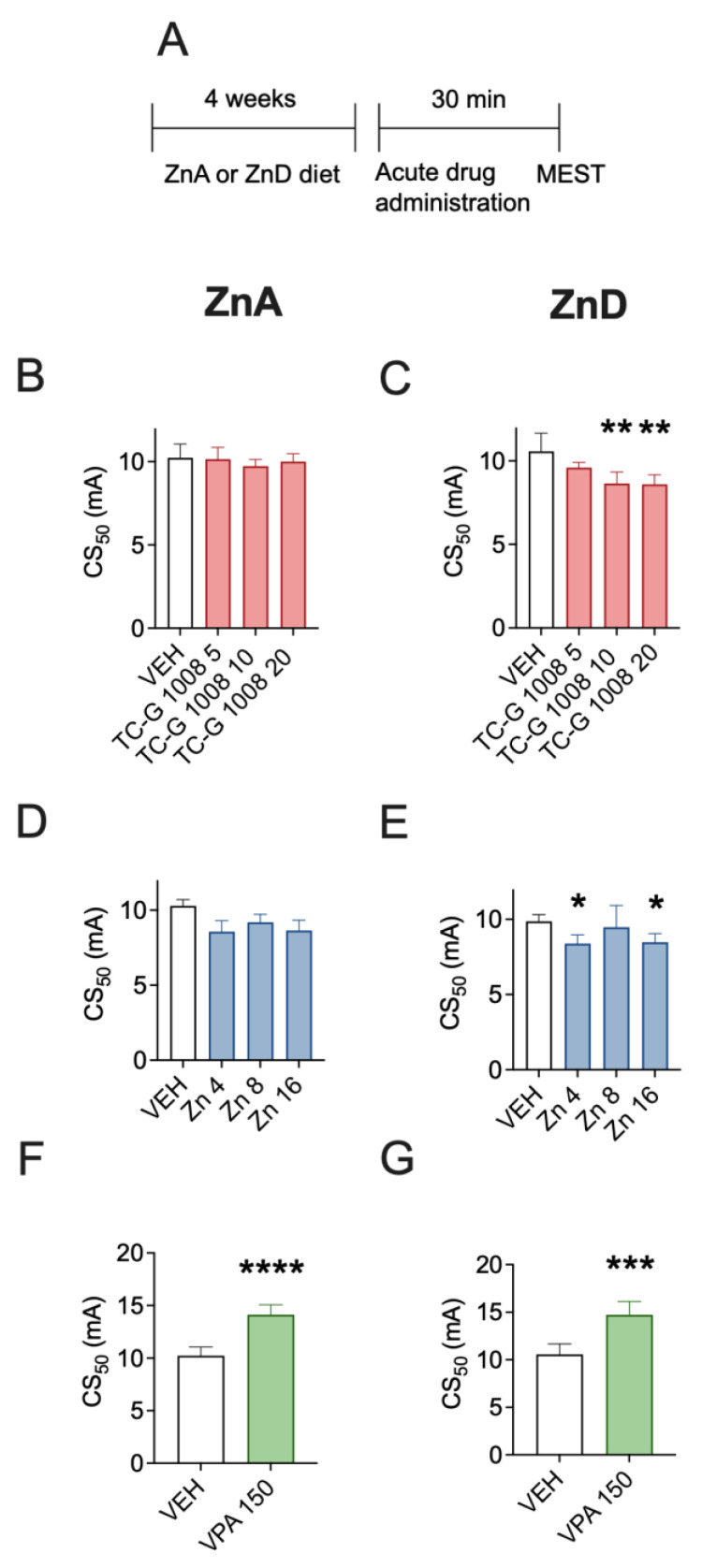
Acute effects of TC-G 1008, zinc chloride (ZnCl_2_), or valproic acid (VPA) on the seizure threshold in the maximal electroshock seizure threshold (MEST) test in mice fed a zinc-adequate diet (ZnA) or a zinc-deficient diet (ZnD) (**A**) Experimental paradigm. Mice were fed the ZnA (50 mg Zn/kg) or the ZnD diet (3 mg Zn/kg) for 4 weeks. Following a 4-week dietary zinc restriction, drugs were administered acutely i.p. The doses of drugs are shown on abscissas in mg/kg. Control mice received vehicle (VEH) (1% Tween 80 in 0.9% NaCl) i.p. 30 min later the MEST test was performed. (**B**–**G**) The results are presented as CS_50_ (in mA) values with upper 95% confidence limits. Data were analyzed by one-way ANOVA and the Dunnett’s multiple comparison test or the Student’s t-test. A total of 20 mice per group were used to determine the seizure threshold. Statistical details: (**B**) F(3,35) = 0.5781, *p* = 0.6333, (**C**) F(3,35) = 6.014, *p* = 0.002, (**D**) F(3,32) = 0.5369, *p* = 0.6604, (**E**) F(3,34) = 5.212, *p* = 0.0045 (B-E, one-way ANOVA), (**F**) t(17) = 6.631, *p* < 0.0001, (**G**) t(18) = 4.953, *p* = 0.0001 (F-G, Student’s t-test). * *p* < 0.05, ** *p* < 0.01, *** *p* < 0.001, **** *p* < 0.0001 vs. VEH group (Dunnett’s multiple comparison test or the Student’s t-test).

PTZ kindling is a chronic model that reflects the process of epilepsy development, i.e., epileptogenesis [29]. Administration of a subthreshold dose of PTZ induces focal seizures at the beginning of the paradigm. The seizures evolve in severity and duration with repeated exposure to PTZ and finally generalized tonic–clonic seizures are triggered by the dose that initially produced focal seizures. The mice were injected i.p. with VEH, TC-G 1008, ZnCl_2_, or VPA on every alternate day during weekdays. 30 min later, they were injected i.p. with a subthreshold dose of PTZ. The dose of PTZ (40 mg/kg) has been established as subthreshold in Swiss Albino mice. The dose of VPA (150 mg/kg) has been established to inhibit epileptogenesis in this model [16,30]. Because PTZ-kindling ultimately produces generalized tonic–clonic seizures, as does MES, the dose of TC-G 1008 was selected based on the outcome of the MEST test. Both doses of TC-G 1008: 10 and 20 mg/kg decreased the seizure threshold in the MEST test (Fig 1C) but in the chronic model we administered 10 mg/kg, as in our previous study [16]. We suspected that administration of the higher dose (20 mg/kg) could produce pronounced seizures too quickly, thus preventing observations of the gradual process, which is the core of the kindling phenomenon. 

Immediately following PTZ injection, mice were placed individually into a transparent box without bedding for 30 min for behavioral observation. The seizure severity of each subject was scored using the modified Racine’s scale: stage 0, no response; stage 1, immobility, ear and facial twitching; stage 2, myoclonic jerks; stage 3, forelimb clonus, stage 4, clonic seizure with rearing and falling; stage 5, generalized clonic seizure with loss of righting reflex; stage 6, tonic fore- and hindlimb extension [16,29]. The mean seizure severity scores were calculated for all experimental groups after each PTZ injection. The model was terminated when mice displayed consecutive stage 5 seizures and were considered fully kindled. Control, non-kindled animals received VEH and were injected with physiological saline instead of PTZ solution. *n* = 15 in each group at the beginning of the paradigm, except the ZnA VPA group, in which *n* = 11. Unequal group sizes at the end of the paradigm resulted from mortality during kindling.

### 2.5. Grip Strength Test 

Skeletal muscular strength was evaluated using the grip strength test [31]. The grip strength apparatus (BioSeb, Chaville, France) consisted of a steel wire grid (8 × 8 cm) connected to an isometric force transducer. The animal was lifted by its tail so that it could grasp the grid with its forepaws. The mouse was then gently pulled backward until it released the grid and the maximal force in newtons (N) exerted by the mouse before losing grip was measured. The procedure was repeated three times and the mean force exerted by each mouse before losing grip was recorded. The mean force was then normalized to body weight and expressed in mN/g ± SEM.

### 2.6. Chimney Test

Motor coordination was evaluated using the chimney test [31]. In this test, the inability of animals to climb backward up through a Plexiglas tube (3 cm, inner diameter × 30 cm, length) within 60 s was an indicator of motor impairment.

### 2.7. Tissue Processing for Biochemical Analysis 

The mice were killed ca. 3 min after MES seizures or 24 h after the completion of the PTZ-kindling model. The brains were rapidly dissected on a cold plate into left and right hemispheres. Left hippocampi (dorsal and ventral) were dissected, immediately frozen on dry ice, and stored at −80 °C until Western blot analysis. The right hemispheres were frozen by liquid nitrogen and were stored at −80 °C until cryo-sectioning. Hippocampal coronal sections (12 µm thick) were prepared from the right hemispheres using cryostat microtome Leica CM 1850 (Leica Biosystems Nussloch GmbH, Nussloch, Germany) and were attached to glass slides (SuperFrost microscope slides, cut edges, Thermo Scientific Menzel Glaser (Waltham, MA, USA). The glass slides were stored at −80 °C until further analysis. The trunk blood was collected into tubes without anti-coagulant. The blood was allowed to clot for 15–20 min and then centrifuged for 10 min at 2000× *g* at 4 °C. The resulting supernatant (serum) was pipetted into tubes that were stored at −80 °C until analysis. Blinding was applied during biochemical analyses.

### 2.8. Inductively Coupled Plasma Optical Emission Spectrometry 

Total zinc concentration in sera was determined by Inductively Coupled Plasma Optical Emission Spectrometry (ICP-OES). Serum samples were defrosted. Serum (200 µL) was transferred to digestion vessels (DigiTUBE SCP SCIENCE 50 mL class A) and mixed with 1.5 mL of 65% (*w*/*w*) Suprapur^®®^ nitric acid (Merck) and 5.0 mL of deionized water. Then vessels were placed in heating blocks (DigiPREP SCP SCIENCE) and were digested for 60 min at 120 ℃. After digestion vessels with solution were left to reach room temperature (RT) and filled with deionized water to 10 mL. The analysis was performed using PlasmaQuant PQ 9000 (Analytik Jena GmbH, Jena, Germany). The following operating conditions of ICP-OES were used: power 1300 W, plasma gas flow 14.0 L/min, auxiliary gas flow 0.50 L/min, nebulizer gas flow 0.60 L/min, sample flow rate 1 mL/min, read time 3s, monitoring direction of the plasma flame was axial. Standard solution for calibration curves of zinc at the concentration of 200 µg/L was prepared by diluting zinc 1000 mg/L standard (PlasmaCAL SCP SCIENCE) with 0.5% (*w*/*w*) nitric acid in deionized water. Analysis line used for zinc quantification was 206.2 nm. 

### 2.9. Laser Ablation Inductively Coupled Plasma Mass Spectrometry (LA-ICP-MS)

Total zinc concentration in hippocampal sections was determined semi-quantitatively by Laser Ablation Inductively Coupled Plasma Mass Spectrometry (LA-ICP-MS). Hippocampal coronal sections (12 µm thick) were thawed and dried at RT in the desiccator and placed in the ablation chamber. The sections were analyzed using a laser ablation (LA) system (LSX-500, CETAC Technologies, Omaha, NE, USA) with a quadrupole inductively coupled plasma mass spectrometer (ICP-MS, 7700x, Agilent, Santa Clara, CA, USA). The instruments were optimized daily with the use of a certified reference material of NIST SRM 610 glass, which included the nebulizer gas flow, ion lens voltage, and power of the plasma generator, and were tuned until reaching the maximal intensity for ^24^Mg^+^, ^115^In^+^, ^238^U^+^, and the oxide ratios of ^232^Th^16^O^+^/^232^Th^+^ < 0.2%, as well as doubly charged ions ^42^Ca^2+^/^42^Ca^+^ < 0.2%. The laser parameters were optimized to completely ablate the thin sections and to obtain measurable signals for the analyzed elements, which required the optimization of the following laser parameters: laser energy, laser spot size, frequency of laser shots, and sample scan rate. The instrumental parameters of LA-ICP-MS were as follows: laser energy 2.7 mJ, spot size 150 µm, ablation frequency 4 Hz, scan rate 300 µm/s, nebulizer gas flow 0.99 L/min, plasma power 1550 W, pulse counting mode, dwell time 100 ms per isotope, measured isotopes ^13^C and ^66^Zn. The laser beam scanned a rectangular area of the sample line by line and always from left to right. The number and width of the ablation lines were set individually for each analyzed sample, with 18 lines on average. The detector recorded a time-resolved signal that was used to create a two-dimensional matrix of data points for each sample. For the statistical evaluation of the measurement data, the region of interest (ROI) containing the hippocampus was marked on maps of the distribution of elements in thin sections of brain. The ROIs were selected by drawing the shape in the imaging software tool based on the photograph of the sample taken prior to the ablation. The signals contained in the ROI were averaged. 

### 2.10. Western Blot 

Hippocampi of mice were homogenized in 2% (*w*/*v*) sodium dodecyl-sulfate solution (SDS) (BioShop Canada Inc.), denatured at 95°C for 10 min and centrifuged at 11,000× *g* at 4 °C for 5 min. The total protein concentration was quantified in the supernatant using a Pierce BCA Protein Assay Kit (Thermo Fisher Scientific, Pierce Biotechnology, Rockford, IL, USA). The samples containing 10 µg of protein were prepared using Novex^®®^ Tris-Glycine SDS Sample Buffer (Thermo Fisher Scientific, Carlsbad, CA, USA) and were resolved on a 4–15% Mini-Protean TGX Precast gels (Bio-Rad Laboratories, Inc., Hercules, CA, USA). The proteins were transferred on a nitrocellulose membrane (BIO-RAD Laboratories, Inc., Hercules, CA, USA). The membranes were blocked for 60 min with 1% (*w*/*v*) blocking reagent from the BM Chemiluminescence WB kit (Mouse/Rabbit) (Roche Diagnostic, Mannheim, Germany). The membranes were then incubated with mouse monoclonal antibody-targeting phosphorylated CREB at Ser 133 (anti-phospho-CREB (Ser133) antibody, clone 10E9, Millipore Cat# 05-667, RRID:AB_309889, at a concentration of 0.5 µg/mL) or rabbit monoclonal antibody-targeting CREB (anti-CREB antibody, Abcam Cat# ab32515, RRID:AB_2292301, at a dilution of 1:1000) or rabbit polyclonal antibody-targeting brain-derived neurotrophic factor (BDNF) (anti-BDNF antibody, Novus Cat# NB100-98682, RRID:AB_1290643, at a dilution of 1:1000), or rabbit polyclonal antibody-targeting tyrosine-phosphorylated tropomyosin receptor kinase B (TrkB) (anti-Trk B phosphorylated (pTyr 816) antibody, Novus Cat# NBP1-03499, RRID:AB_1522601, at a concentration of 10 µg/mL), or rabbit monoclonal antibody-targeting TrkB (anti-TrkB antibody, Abcam Cat# ab187041, RRID:AB_2892613, at a dilution of 1:5000), or β-actin (β-actin antibody, mouse monoclonal clone AC-15, purified from hybridoma cell culture, Sigma-Aldrich Cat# A1978, RRID:AB_476692, at a concentration of 0.5 µg/mL) at 2–8°C overnight. The dilutions of primary antibodies were prepared using 0.5% (*w*/*v*) blocking solution from the BM Chemiluminescence WB kit (Mouse/Rabbit) (Roche Diagnostic, Mannheim, Germany). They were stored at 2–8 °C and were reused up to two times. On the next day, after washing with TBST 3 × 10 min, the membranes were incubated for 30 min with horseradish peroxidase-linked (HRP-linked) secondary antibody from the BM Chemiluminescence WB kit (Mouse/Rabbit), at the concentration of 40 mU/mL, or the anti-mouse IgG, HRP-linked, Cell Signaling Cat# 7076, RRID:AB_330924, at the dilution of 1:1000 under constant shaking at RT. The dilutions of secondary antibodies were always prepared fresh. After incubation with secondary antibodies, the membranes were washed with TBST 3 × 10 min. Secondary antibodies were detected using a BM Chemiluminescence WB kit (Mouse/Rabbit). The protein bands were visualized with the ChemiDoc Imaging System (Bio-Rad Laboratories, Inc., Hercules, CA, USA). The density of each protein band was analyzed using imaging software (Image Lab, Bio-Rad Laboratories, Inc., Hercules, CA, USA) and was normalized by the optical density of the corresponding β-actin band. 

### 2.11. Data and Statistical Analysis

Data were analyzed using GraphPad Prism v. 9.4.1 (GraphPad Software, San Diego, CA, USA) or STATISTICA v. 13.3 (TIBCO Software Inc., Palo Alto, CA, USA). No statistical method was used to predetermine the sample size. Data were screened for outliers using the Grubbs’s test (https://www.graphpad.com/quickcalcs/Grubbs1.cfm, accessed on 15 October 2022) and outliers were excluded from the analyses. MEST test was analyzed by the unpaired Student’s *t*-test or one-way analysis of variance (ANOVA) and the Dunnett’s multiple comparison test. Seizure severity during PTZ-kindling was analyzed by two-way repeated measures ANOVA and the Dunnett’s multiple comparison test. Percentage of fully kindled mice, mortality, and motor coordination after the last injection of PTZ in the kindling model were analyzed by the Chi square test (https://www.graphpad.com/quickcalcs/contingency1, accessed on 15 October 2022). Neuromuscular strength after the last injection of PTZ was analyzed by the one-way ANOVA and the Dunnett’s multiple comparison test. For Western blot, ICP-OES, or LA-ICP-MS each sample was run in at least duplicate. The results of Western blot and ICP-OES were analyzed using one-way ANOVA and the Dunnett’s multiple comparison test. In all cases, the Dunnett’s multiple comparison test was used only when F was statistically significant. The results are presented as the mean ± SEM. *p* < 0.05 was considered statistically significant with 95% confidence. 

## 3. Results

### 3.1. Acute Effects of TC-G 1008, ZnCl_2_, or VPA on the Seizure Threshold in the MEST Test in Mice Fed the ZnA or ZnD Diet 

First, we tested whether the acute effects of GPR39 agonists depend on zinc supply. The mice were fed the ZnA diet of 50 mg Zn/kg or the ZnD diet of 3 mg Zn/kg for 4 weeks. Following 4 weeks of dietary zinc restrcition, the acute effects of drugs on the seizure threshold were examined in the MEST test (Figure 1A). TC-G 1008, at doses of 5, 10, or 20 mg/kg, did not significantly affect the seizure threshold in mice fed the ZnA diet (Figure 1B). Similarly, ZnCl_2_, at doses of 4, 8, or 16 mg Zn/kg did not significantly affect the seizure threshold in mice fed the ZnA diet (Figure 1D). Both TC-G 1008 and ZnCl_2_ decreased the seizure threshold in mice fed the ZnD diet. TC-G 1008 decreased the seizure threshold in ZnD mice at doses of 10 or 20 mg/kg (Figure 1C), while ZnCl_2_ exerted such an effect at doses of 4 or 16 mg Zn/kg (Figure 1E). In contrast, VPA (150 mg/kg) significantly increased the seizure threshold in the MEST test in both mice fed the ZnA (Figure 1F) and ZnD (Figure 1G) diet for 4 weeks. 

### 3.2. Acute Effects of TC-G 1008, ZnCl_2_, or VPA on MES Seizures in Mice Fed the ZnA or ZnD Diet—Biochemical Analyses in Serum and Hippocampus

**Figure 2:**
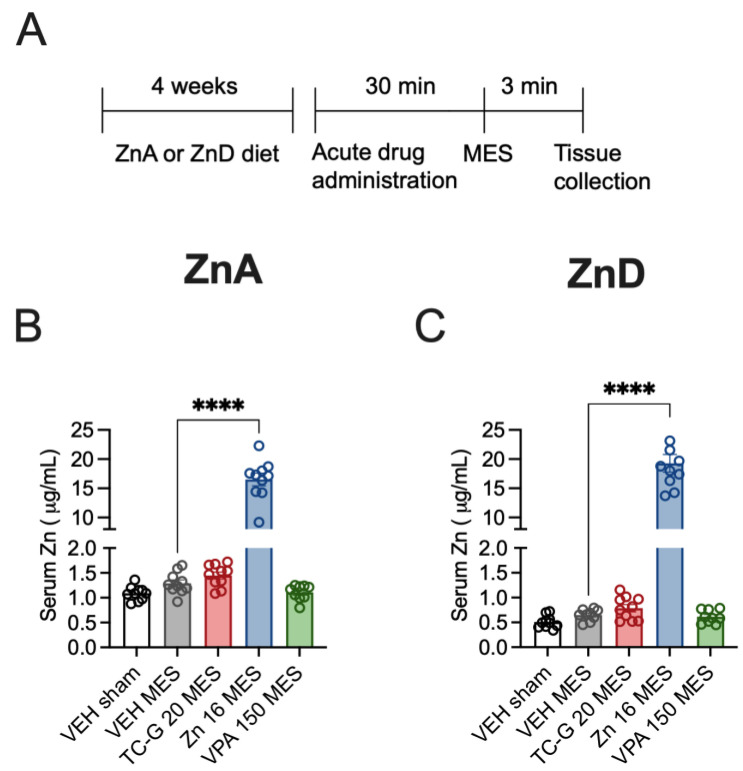
Acute effects of TC-G 1008 (TC-G) (20 mg/kg), ZnCl_2_ (16 mg Zn/kg) or VPA (150 mg/kg) and maximal electroshock (MES) seizures on total zinc concentration in the sera of mice fed the ZnA or ZnD diet. (**A**) Experimental paradigm. Mice were fed the ZnA or ZnD diet for 4 weeks. Following a 4-week dietary zinc restriction, drugs were administered acutely i.p. Control mice received VEH i.p. 30 min later, seizures were generated by supramaximal current intensity of 50 mA. Non-stimulated (sham) mice received VEH but they did not receive the electrical stimulus. The serum samples were obtained ca. 3 min after MES. Total serum zinc concentration was measured by Inductively Coupled Plasma Optical Emission Spectrometry (ICP-OES). (**B**,**C**) Data are expressed as means ± SEM. They were analyzed by one-way ANOVA and the Dunnett’s multiple comparison test. *n* = 9–10 in each group. Statistical details: (**B**) F(4,44) = 191.4, *p* < 0.0001, (**C**) F(4,43) = 142.1, *p* < 0.0001 (B-C one-way ANOVA). **** *p* < 0.0001 vs. VEH MES group (Dunnett’s multiple comparison test).

The biochemical analyses were performed ca. 3 min after MES seizures, which were induced after 4 weeks of dietary zinc restriction (Figure 2A). The mean serum zinc concentration in sham mice fed the ZnA diet for 4 weeks was 1.09 µg/mL (Figure 2B). The mean serum zinc concentration in sham mice fed the ZnD diet for 4 weeks was 0.51 µg/mL (Figure 2C). Diurnal and post-prandial variations in serum zinc have been observed in humans [32]; moreover, diurnal differences in zinc homeostasis protein (metallothionenin) have been demonstrated in the blood of mice [33]. It is possible that the absolute values of zinc concentration measured in serum of mice would be different than those reported above after sampling at different time. Nevertheless, the serum zinc concentration declined by 53% after 4 weeks of dietary zinc restriction, which is consistent with previous data [34,35,36]. The mean serum zinc concentration in mice fed the ZnA diet that underwent MES seizures was 1.3 µg/mL (Figure 2B), while the concentration in ZnD mice that underwent MES seizures was 0.65 µg/mL (Figure 2C). Hence, acute MES seizures did not significantly affect total serum zinc concentration in either mice fed the ZnA (Figure 2B) or ZnD diet (Figure 2C). 

Acute treatment with TC-G 1008 (20 mg/kg) or VPA (150 mg/kg) did not significantly affect total serum zinc concentration in mice fed the ZnA diet that underwent MES seizures, compared to VEH (Figure 2B). Similarly, acute treatment with these compounds did not significantly affect serum zinc concentration in ZnD mice that underwent MES seizures, compared to VEH (Figure 2C). In contrast, administration of a single dose of ZnCl_2_ (16 mg Zn/kg), followed by exposure to MES, markedly and significantly increased serum zinc concentration, compared to mice that received VEH and MES, in both mice fed the ZnA (Figure 2B) and the ZnD (Figure 2C) diet. As MES seizures were induced 30 min after acute drug administration, a robust and rapid increase in serum zinc was observed after i.p. administration of ZnCl_2_ but not after treatment with TC-G 1008 or VPA.

**Figure 3:**
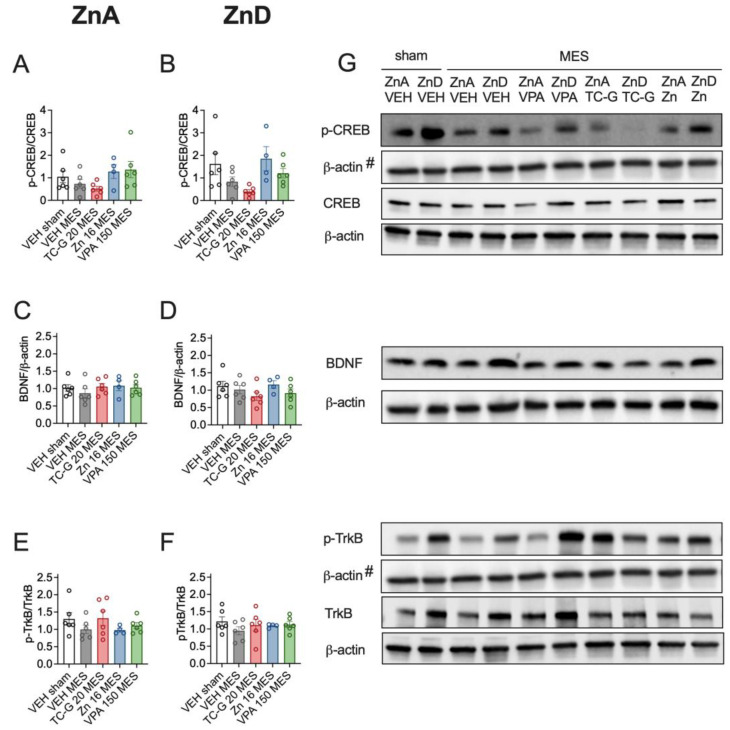
Acute effects of TC-G 1008 (TC-G) (20 mg/kg), ZnCl_2_ (16 mg Zn/kg), or VPA (150 mg/kg) and MES seizures on the relative expression of proteins: phosphorylated CREB (p-CREB), total CREB, BDNF, phosphorylated TrkB (p-TrkB) and TrkB in the hippocampi of mice fed the ZnA or ZnD diet. Hippocampal samples were obtained ca. 3 min after MES. (**A**–**F**) Data are expressed as means ± SEM of p-CREB/CREB or BDNF/β-actin or p-TrkB/TrkB ratio. They were analyzed by one-way ANOVA. *n* = 4–6 in each group. Statistical details: (**A**) F(4,23) = 1.100, *p* = 0.3805, (**B**) F(4,23) = 0.6353, *p* = 0.6425, (**C**) F(4,23) = 0.5934, *p* = 0.6709, (**D**) F(4,23) = 1.218, *p* = 0.3306, (**E**) F(4,23) = 1.100, *p* = 0.3805, (**F**) F(4,23) = 0.6353, *p* = 0.6425 (A-F one-way ANOVA). (**G**) Representative blots of *p*-CREB (Ser 133), CREB (~46 kDa), BDNF (~14 kDa), *p*-TrkB (Tyr 816), TrkB (~140 kDa) and β-actin (~42 kDa) in the hippocampi of mice. ^#^p-CREB and p-TrkB come from the same blot, thus sharing the corresponding β-actin band.

Moreover, MES seizures did not significantly affect the expression of p-CREB/CREB, BDNF, or p-TrkB/TrkB in the hippocampus of mice fed either the ZnA (Figure 3A,C,E) or the ZnD diet for 4 weeks (Figure 3B,D,F). In addition, there were no significant differences in the expression of the selected proteins after the administration of single doses of TC-G 1008 (20 mg/kg), ZnCl_2_ (16 mg Zn/kg), or VPA (150 mg/kg) in mice that underwent MES and were fed either the ZnA (Figure 3A,C,E) or the ZnD diet (Figure 3B,D,F) for 4 weeks. 

### 3.3. Chronic Effects of TC-G 1008, ZnCl_2_, or VPA on PTZ-Induced Epileptogenesis in Mice Fed the ZnA or ZnD Diet

**Figure 4:**
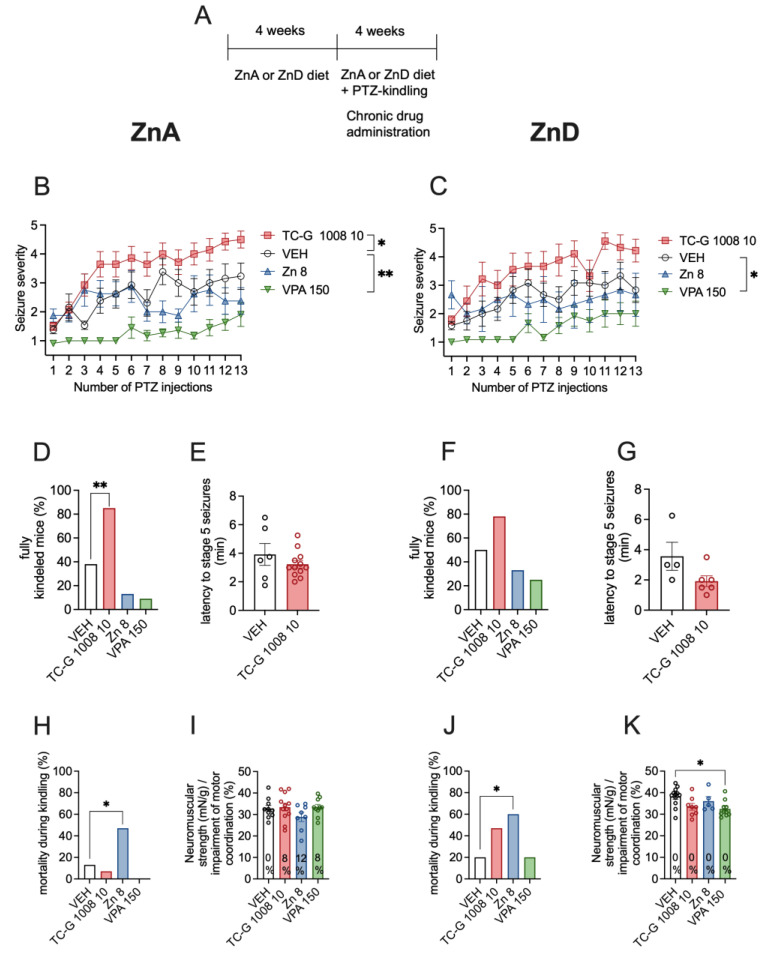
Chronic effects of TC-G 1008 (10 mg/kg), ZnCl_2_ (8 mg Zn/kg), or VPA (150 mg/kg) on pentylenetetrazole (PTZ)-induced epileptogenesis in mice fed the ZnA or ZnD diet. (**A**) Experimental paradigm. Mice were fed the ZnA or ZnD diet for 4 weeks. Following a 4-week dietary zinc restriction, PTZ-kindling model of epilepsy began. TC-G 1008 (10 mg/kg), ZnCl_2_ (8 mg Zn/kg), VPA (150 mg/kg), or VEH were injected i.p. once daily on every alternate day during weekdays. 30 min later, PTZ (40 mg/kg) was injected i.p. Immediately after each PTZ injection the mice were subjected to an evaluation of behavioral seizures, which lasted 30 min. The total number of PTZ injections was 13. The ZnA or ZnD diet was continued during this chronic paradigm. (**B**,**C**) Data are expressed as means ± SEM of seizure severity after each PTZ injection. They were analyzed by two-way repeated measures ANOVA and a Dunnett’s multiple comparison test. (**D**,**F**) The percentage of mice displaying consecutive stage 5 seizures (fully kindled mice) after the last PTZ injection. Data were analyzed by the Chi square test. (**E**,**G**) Data are expressed as means ± SEM of latency to stage 5 seizures after the last PTZ injection. They were analyzed by the Student’s t-test. (**H**, **J**) Mortality during kindling was analyzed by the Chi square test after the last PTZ injection. (**I**,**K**) Neuromuscular strength (assessed in the grip strength test) and motor coordination (assessed in the chimney test, expressed as % of mice that displayed motor impairment in this test) after the last PTZ injection. Neuromuscular strength was analyzed by one-way ANOVA and a Dunnett’s multiple comparison test. Motor coordination was analyzed by Chi square test. *n* at the end of kindling procedure: *n* = 13 ZnA VEH, *n* = 14 ZnA TC-G 1008 10 mg/kg, *n* = 8 ZnA Zn 8 mg/kg, *n* = 11 ZnA VPA 150 mg/kg, *n* = 12 ZnD VEH, *n* = 9 ZnD TC-G 1008 10 mg/kg, *n* = 6 ZnD Zn 8 mg/kg, *n* = 12 ZnD VPA 150 mg/kg. Unequal group sizes result from mortality during kindling. Statistical details: (**B**) drug (F(3,42) = 15.24, *p* < 0.0001), time (F(8.013, 336.5) = 10.33, *p* < 0.0001), drug × time (F(36,504) = 1.870, *p* = 0.002) (two-way repeated measures ANOVA); (**C**) drug (F(3,35) = 8.557, *p* = 0.0002), time (F(6.220, 217.7) = 5.922, *p* < 0.0001), drug × time (F(36,420) = 0.9082, *p* = 0.6244) (two-way repeated measures ANOVA), (**D**) *p* = 0.01 (TC-G 1008), *p* = 0.2 (ZnCl_2_), *p* = 0.09 (VPA) (Chi square test), (**E**) t(16) = 1.056, *p* = 0.3068 (Student’s t-test), (**F**) *p* = 0.19 (TC-G 1008), *p* = 0.5 (ZnCl_2_), *p* = 0.21 (VPA) (Chi square test), (**G**) t(8) = 1.927, *p* = 0.0901 (Student’s t-test), (**H**) *p* = 0.37 (TC-G 1008), *p* = 0.05 (ZnCl_2_), *p* = 0.21 (VPA) (Chi square test), (**I**) neuromuscular strength: F(3,36) = 1.428, *p* = 0.25 (one-way ANOVA), motor coordination: *p* = 0.35 (TC-G 1008), *p* = 0.25 (ZnCl_2_), *p* = 0.31 (VPA) (Chi square test), (**J**) *p* = 0.12 (TC-G 1008), *p* = 0.03 (ZnCl_2_), *p* = 1 (VPA) (Chi square test), (**K**) neuromuscular strength: F(3,31) = 3.799, *p* = 0.02 (one-way ANOVA). * *p* < 0.05, ** *p* < 0.01 vs. VEH group (Dunnett’s multiple comparison test or the Chi square test).

Next, we tested whether the chronic effects of GPR39 agonists depend on zinc supply. The chronic model of epilepsy (PTZ-induced kindling) began after the initial 4 weeks of dietary zinc restriction (Figure 4A). In this model, TC-G 1008 (10 mg/kg) significantly increased the maximal seizure severity, compared to VEH, in mice fed the ZnA diet (Figure 4B), but not in mice fed the ZnD diet (Figure 4C). VPA (150 mg/kg) decreased the maximal seizure severity in both mice fed the ZnA (Figure 4B) and ZnD (Figure 4C) diet. ZnCl_2_ (8 mg Zn/kg) did not significantly affect this parameter in mice fed either the ZnA (Figure 4B) or the ZnD (Figure 4C) diet. 

After 13 injections of PTZ (40 mg/kg), the percentage of fully kindled mice among those fed the ZnA diet was 38% of mice that received VEH, 85% of mice that were administered with TC-G 1008 (10 mg/kg), 13% of mice supplemented with ZnCl_2_ (8 mg Zn/kg), and 9% of mice that were treated with VPA (150 mg/kg). Thus, TC-G 1008 significantly increased the percentage of fully kindled mice that were fed the ZnA diet (Figure 4D). In mice fed the ZnD diet, the percentage of fully kindled mice was 50% of those that received VEH, 78% of mice that were administered with TC-G 1008 (10 mg/kg), 33% of mice that were supplemented with ZnCl_2_ (8 mg Zn/kg), and 25% of mice that were treated with VPA (150 mg/kg) (Figure 4F). Taken together, the ZnD diet abolished the chronic effects of TC-G 1008 on the maximal seizure severity and percentage of fully kindled mice in this model. However, the mean latency to stage 5 seizures after the last injection of PTZ in mice that received TC-G 1008 was 3.25 min in those fed the ZnA diet (Figure 4E), while it was 1.9 min in ZnD mice (Figure 4G). The decrease in the mean latency to stage 5 seizures after repeated administration of TC-G 1008 was not significant in both cases. 

The mortality of kindled ZnD mice that received TC-G 1008 was increased, though not significantly (Figure 4J). After 13 injections of PTZ (40 mg/kg), the mortality of ZnD mice was 20% of those that received VEH, 47% of mice that were administered with TC-G 1008 (10 mg/kg), 60% of mice that were supplemented with ZnCl_2_ (8 mg Zn/kg), and 20% of mice that were treated with VPA (150 mg/kg) (Figure 4J). Thus, ZnCl_2_ supplementation for >4 weeks significantly increased mortality in kindled ZnD mice (Figure 4J)_._

ZnCl_2_ administration during kindling increased mortality also in mice fed the ZnA diet (Figure 4H). Although ZnCl_2_ did not significantly affect the maximal seizure severity (Figure 4B,C), it decreased the percentage of fully kindled mice, as did VPA (the decrease in the maximal seizure severity was not significant in cases of both drugs) (Figure 4D,F). However, the observation on increased mortality precludes this dose and form of zinc (ZnCl_2_, 8 mg Zn/kg, i.p.) from being regarded as an anti-epileptogenic treatment. To reduce mortality kindling was terminated after 13 injections of PTZ (40 mg/kg). 

After 13 injections of PTZ (40 mg/kg), none of the drugs significantly affected the motor coordination in mice fed either the ZnA (Figure 4I) or ZnD (Figure 4K) diet. None of the drugs significantly affected the neuromuscular strength of mice fed the ZnA diet (Figure 4I). Moreover, TC-G 1008 (10 mg/kg) and ZnCl_2_ (8 mg Zn/kg) did not significantly affect the neuromuscular strength in ZnD mice, while VPA (150 mg/kg) decreased this parameter in those mice (Figure 4K). 

### 3.4. Chronic Effects of TC-G 1008, ZnCl_2_, or VPA on PTZ-Induced Epileptogenesis in Mice Fed the ZnA or ZnD Diet—Biochemical Analyses in Serum and Hippocampus

**Figure 5:**
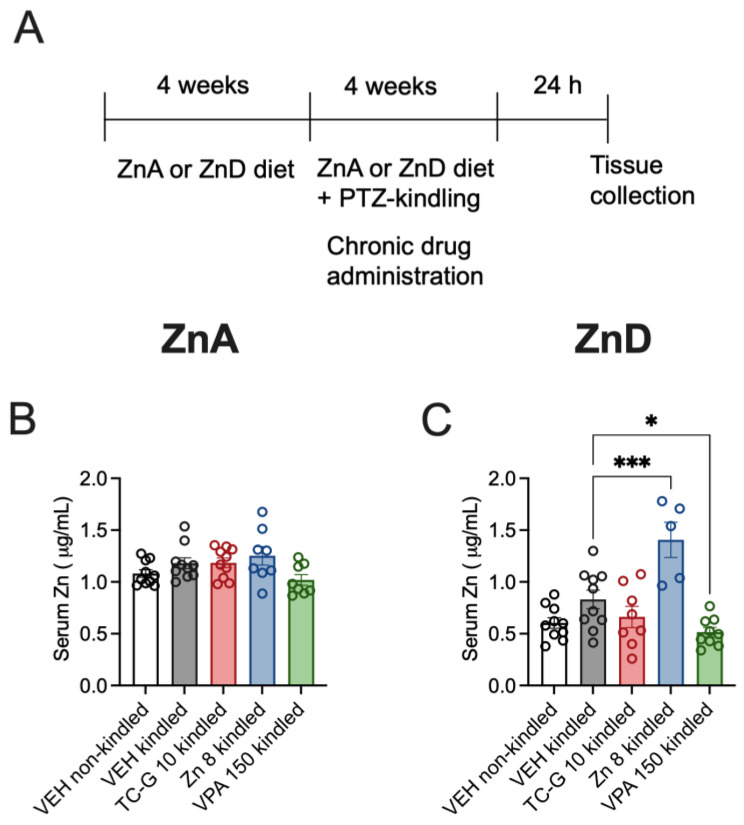
Chronic effects of TC-G 1008 (TC-G) (10 mg/kg), ZnCl_2_ (8 mg Zn/kg), or VPA (150 mg/kg) and the PTZ-kindling model of epilepsy on the total zinc concentration in the sera of mice fed the ZnA or ZnD diet. (**A**) Experimental paradigm. Mice were fed the ZnA or ZnD diet for 4 weeks. Following a 4-week dietary zinc restriction, the PTZ-kindling model of epilepsy began. The model consisted of 13 injections of PTZ (40 mg/kg). Non-kindled mice received VEH and physiological saline, instead of PTZ. The ZnA or ZnD diet was continued during this chronic paradigm. The serum samples were obtained 24 h after the last injection of PTZ. Total serum zinc concentration was measured by ICP-OES. (**B**,**C**) Data are expressed as means ± SEM. They were analyzed by one-way ANOVA and the Dunnett’s multiple comparison test. *n* = 5–10 in each group. Statistical details: (**B**) F(4,41) = 2.564, *p* = 0.0525 (**C**) F(4,37) = 12.15, *p* < 0.0001 (B-C one-way ANOVA). * *p* < 0.05, *** *p* < 0.001 vs. VEH kindled group (Dunnett’s multiple comparison test).

The biochemical analyses were performed after the completion of the PTZ-kindling model, which began after the initial 4 weeks of dietary zinc restriction and consisted of 13 injections of PTZ (40 mg/kg), during which the diet was continued. The analyses were thus performed after >8 weeks of dietary zinc deficiency, including >4 weeks of drug administration (Figure 5A). 

The mean serum zinc concentration in non-kindled mice fed the ZnA diet for >8 weeks was 1.08 µg/mL (Figure 5B). The mean serum zinc concentration in non-kindled mice fed the ZnD diet for 8 weeks was 0.60 µg/mL (Figure 5C). Thus, the decline by >50% (55%) in total serum zinc concentration was maintained during prolonged dietary zinc restriction. The mean serum zinc concentration in kindled mice fed the ZnA diet was 1.18 µg/mL (Figure 5B), while the concentration in kindled ZnD mice was 0.83 µg/mL. (Figure 5C). Hence, the kindling procedure did not significantly affect serum zinc concentration in mice fed either the ZnA or ZnD diet. 

Chronic administration of TC-G 1008 (10 mg/kg), ZnCl_2_ (8 mg Zn/kg), or VPA (150 mg/kg) did not significantly affect total serum zinc concentration in kindled mice fed the ZnA diet, compared to VEH (Figure 5B). However, chronic treatment with ZnCl_2_ (8 mg Zn/kg) or VPA (150 mg/kg) affected serum zinc concentration in kindled mice fed the ZnD diet: treatment with ZnCl_2_ increased, while treatment with VPA decreased this parameter, compared to VEH (Figure 5C). The mean serum zinc concentration in kindled mice fed the ZnA diet and supplemented with ZnCl_2_ for >4 weeks was 1.25 µg/mL, while it was 1.41 µg/mL in kindled ZnD mice supplemented with ZnCl_2._ Hence, zinc supplementation significantly increased serum zinc in kindled ZnD mice (Figure 5C) but not in kindled ZnA mice (Figure 5B). As the serum samples were obtained 24 h after completion of the kindling procedure, the increase in serum zinc following chronic treatment with ZnCl_2_, measured 24 h after injection (Figure 5B,C) was much less robust than the increase observed 30 min after treatment (Figure 2B,C). In contrast to ZnCl_2_ and VPA, chronic administration of TC-G 1008 (10 mg/kg) did not significantly affect serum zinc in kindled ZnD mice, compared to VEH (Figure 5C). 

**Figure 6:**
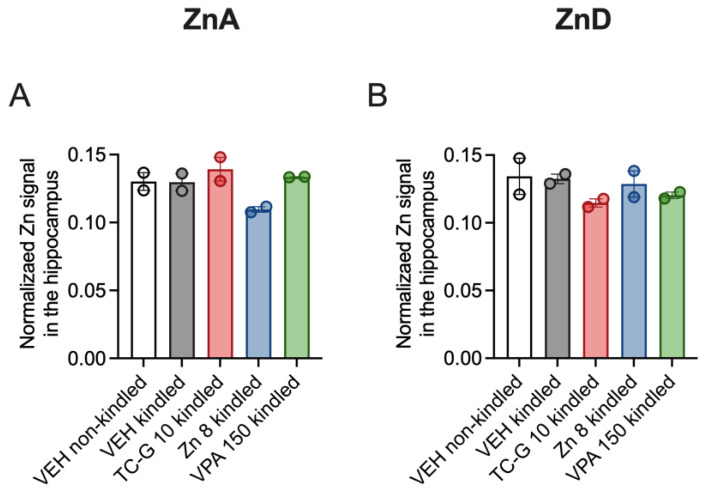
Chronic effects of TC-G 1008 (TC-G) (10 mg/kg), ZnCl_2_ (8 mg Zn/kg), or VPA (150 mg/kg) and PTZ-kindling model of epilepsy on total zinc concentration in the hippocampi of mice fed the ZnA (**A**) or ZnD (**B**) diet. Brain samples were obtained 24 after the last injection of PTZ. Total zinc concentration was measured in hippocampal coronal sections using Inductively Coupled Plasma Mass Spectrometry (ICP-MS). Data are presented as means ± SEM of three replicates per mouse brain. Measurements for 2 brains per group are shown.

Due to the mixed results of previous studies examining the effects of a 4-week dietary zinc restriction on total brain zinc [36,37,38], we did not suspect changes in hippocampal zinc after 4 weeks of the ZnD diet administration. We therefore performed the ICP-MS analysis of hippocampal zinc after the completion of the PTZ-kindling model, i.e., after >8 weeks of dietary zinc deficiency (Figure 6). The results were not analyzed statistically due to small sample sizes (two mice per group). The mean normalized zinc signal in the hippocampus was 0.13 in both mice fed the ZnA and ZnD diet for >8 weeks. Furthermore, we did not observe a tendency towards increased hippocampal zinc in ZnCl_2_ supplemented mice that displayed increased serum zinc. 

**Figure 7:**
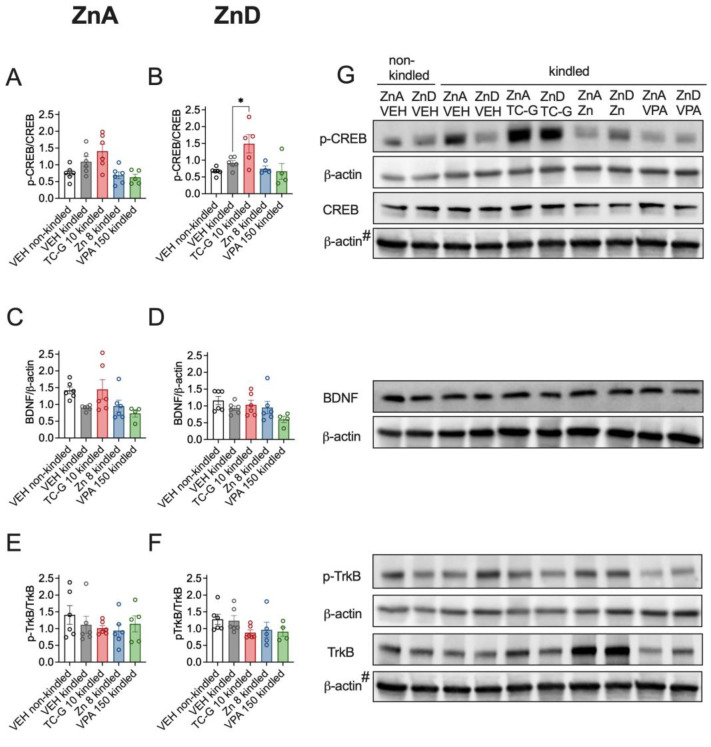
Chronic effects of TC-G 1008 (TC-G) (10 mg/kg), ZnCl_2_ (8 mg Zn/kg) or VPA (150 mg/kg) and PTZ-kindling model of epilepsy on the relative expression of proteins: p-CREB, CREB, BDNF, p-TrkB and TrkB in the hippocampi of mice fed the ZnA or ZnD diet. Hippocampal samples were obtained 24 h after the last injection of PTZ. (**A**–**F**) Data are expressed as means ± SEM of p-CREB/CREB or BDNF/β-actin or p-TrkB/TrkB ratio. They were analyzed by one-way ANOVA and the Dunnett’s multiple comparison test. *n* = 4–6 in each group. Statistical details: (**A**) F(4,24) = 5.636, *p* = 0.0024, (**B**) F(4,20) = 4.883, *p* = 0.0065, (**C**) F(4,23) = 3246, *p* = 0.0299, (**D**) F(4,23) = 1998, *p* = 0.1284, (**E**) F(4,24) = 0.6798, *p* = 0.6127, (**F**) F(4,22) = 1.547, *p* = 0.2236 (A-F one-way ANOVA). * *p* < 0.05 vs. VEH kindled group (Dunnett’s multiple comparison test). (**G**) Representative blots of p-CREB (Ser 133), CREB (~46 kDa), BDNF (~14 kDa), p-TrkB (Tyr 816), TrkB (~140 kDa), and β-actin (~42 kDa) in the hippocampi of mice. ^#^CREB and TrkB come from the same blot, thus sharing the corresponding β-actin band.

In addition, kindling induced by 13 injections of PTZ (40 mg/kg) did not significantly affect the expression of p-CREB/CREB, BDNF or p-TrkB/TrkB in the hippocampus of mice fed the ZnA diet of 50 mg Zn/kg (Figure 7A,C,E) or the ZnD diet (Figure 7B,D,F). Moreover, the examined compounds did not significantly affect the expression of the selected proteins in kindled mice that were fed the ZnA diet (Figure 7A,C,E). However, TC-G 1008 (10 mg/kg), but not ZnCl_2_ or VPA (150 mg/kg) increased the p-CREB/CREB ratio in the hippocampus of kindled ZnD mice (Figure 7B). Furthermore, there was a tendency towards increased pCREB/CREB (Figure 7A) and BDNF (Figure 7C) protein level in the hippocampus of kindled mice that were fed the ZnA diet and treated with TC-G 1008 (10 mg/kg). There were no significant changes in the expression of BDNF (Figure 7D) or p-TrkB/TrkB ratio (Figure 7F) in the hippocampus of kindled ZnD mice that received any of the drugs.

## 4. Discussion

We previously assessed the effects of the GPR39 agonist, TC-G 1008, on the seizure threshold in the MEST test and on PTZ-induced epileptogenesis in mice fed the diet containing 25 mg Zn/kg [16]. Here, we modified the diets and used the following: ZnA of 50 mg Zn/kg and ZnD of 3 mg Zn/kg. Our previous and present study collectively showed that TC-G 1008 decreased the seizure threshold in the MEST test in mice fed the ZnD diet of 3 mg Zn/kg and in mice fed the diet of 25 mg Zn/kg, but not in mice that received the diet of 50 mg Zn/kg. Conversely, TC-G 1008 facilitated PTZ-induced epileptogenesis in mice fed the diet of 50 mg Zn/kg and 25 mg/kg, while it did not significantly affect epileptogenesis in ZnD mice. Thus, the acute effects of TC-G 1008 on the seizure threshold and the chronic effects on epileptogenesis were differentially regulated by dietary zinc supply. Decreasing the amount of zinc in a diet was associated with decreased seizure threshold, while increasing dietary zinc was accompanied by enhanced epileptogenesis. 

Our study attempted to verify whether the effects of TC-G 1008 depend on zinc status. We hypothesized that the effects would differ under the condition of zinc deficiency, when there would be a reduced supply of the presumed endogenous agonist of GPR39—the essential element zinc. The in vivo inhibitory effect of dietary zinc restriction on the activity of TC-G 1008 is supported by observations that the compound did not significantly affect the maximal seizure severity and percentage of fully kindled mice in those fed the ZnD diet, while it enhanced these parameters in mice fed the ZnA diet. The ZnD diet abolished the chronic effects of TC-G 1008 on these parameters, thereby showing that zinc modulates the effects of TC-G 1008 not only in vitro [19] but also in vivo. 

We previously found that TC-G 1008 aggravated PTZ-induced epileptogenesis by acting selectively at GPR39 [16]. The ZnD diet decreased the expression of GPR39 at the protein level in the hippocampus [12,25]. Decreased GPR39 expression in mice fed the ZnD diet, and thus a lower availability of the target for TC-G 1008 in the chronic model, might have led to milder effects of TC-G 1008 on epileptogenesis observed in ZnD mice. However, the latency to stage 5 seizures after the last injection of PTZ and the compound in the kindling model was 3.2 min and 1.9 min in mice fed the ZnA and ZnD diet, respectively. 

TC-G 1008 is the most potent among the currently available agonists at GPR39 [20]. It binds, however, to the serotonin 5-HT1A receptor [19]. We found non-selective effects of TC-G 1008 on the expression of p-CREB/CREB [16] and TRPM7 [39] proteins in the hippocampus. In the MEST test, we did not exclude the involvement of other targets such as 5-HT1A receptor in the activity of TC-G 1008 [16]. In this study, the acute effects of TC-G 1008 on the seizure threshold in the MEST test were observed in mice fed the ZnD diet but not in mice fed the ZnA diet. It is possible that in the MEST test targets other than GPR39 play a role. That may underlie the distinct regulation of the effects of TC-G 1008 in the acute test/the chronic model by dietary zinc. Another possibility is that a particular signaling pathway downstream of GPR39 [18,19] is involved in the effect of TC-G 1008 in the MEST test.

Sunuwar et al. [40] verified whether the effects of GPR39 KO depend on zinc supply. They used a “high zinc” diet of 70 ppm. Noteworthy, their diet (Harlan, Teklad global 18) is considered as a standard diet for rodents. Moreover, the diet that we used in our previous study (Murigran, Agropol S.J.), containing 25 mg Zn/kg, is considered as standard. Notably, the zinc requirement for rats is 12 mg Zn/kg of diet [41] and the recommendation of the American Institute of Nutrition-93 diet is 30 mg Zn/kg [42]. Nevertheless, the amount of zinc in diets labeled by manufactures as standard varies between 25 (our previous study), 50 (Altromin GmbH), and 70 (Harlan) mg Zn/kg. In contrast, the zinc content of a zinc supplemented diet is several hundred mg Zn/kg (e.g., 248 mg Zn/kg [43], 246 mg Zn/kg [44] or 300 mg Zn/kg [45]).

Sunuwar et al. [40] also utilized a “moderate” diet of 30 ppm zinc and a ZnD diet of 3 ppm zinc. They observed that when the mice were fed the diet of 70 ppm or 30 ppm, intestinal fluid secretion due to cholera toxin administration was increased in GPR39 KO mice, compared to WT mice. However, when mice were fed the diet of 3 ppm there was no significant difference in intestinal fluid secretion between GPR39 KO and WT mice. Hence, the ZnD diet abolished the effects of GPR39 KO in a model of diarrhea, emphasizing the role of zinc homeostasis in the function of the GPR39 receptor. Hitherto, the relationship between the ZnD diet and the function of the GPR39 receptor [40]/the activity of the agonist, TC-G 1008, has been shown. 

In this study, the percentage of fully kindled mice that received TC-G 1008 during PTZ-kindling was 85 and 78% of mice that were fed the ZnA and the ZnD diet, respectively. In our previous study, the percentage of fully kindled mice that received TC-G 1008 during PTZ-kindling was 87% in the case of Swiss Albino mice and 83.3% of WT C57BL/6/Tar x CBA/Tar mice [16]. TC-G 1008 thus consistently aggravated epileptogenesis in the PTZ-kindling model. Furthermore, we replicated the findings that TC-G 1008 decreased the seizure threshold in the MEST test [16]. Replication was of crucial importance because the prevailing hypothesis was that GPR39 activation is a new therapeutic strategy for treating acute seizures/epilepsy [15], while our results are contrary to this hypothesis. 

The effects of TC-G 1008 on PTZ-epileptogenesis were mediated selectively by GPR39, but we showed the non-selective activity of TC-G 1008 upon GPR39 on CREB activation in the hippocampus. We observed a marked increase in the p-CREB/CREB ratio in the hippocampus of GPR39 KO mice subjected to the PTZ-kindling model and chronic treatment with TC-G 1008 (10 mg/kg) but not in WT mice [16]. The 5-HT1A receptor, which is an additional target for TC-G 1008 [19], modulates CREB levels. The 5-HT1A is coupled to the G_i_ protein, which inhibits adenylate cyclase (AC) and CREB, but activation of AC was found after activation of 5-HT1A in hippocampal cells. Indeed, the activation of 5HT1A results in the activation of AC type II, which leads to CREB phosphorylation [46]. As TC-G 1008 binds to the 5-HT1A [19], our data suggest that in the absence of GPR39, TC-G 1008 increases CREB phosphorylation via this subtype of serotonin receptor [16].

Why would the presence of GPR39 in WT mice prevent CREB activation [16]? The reason may be the formation of receptor dimers or trimers [47]. 5-HT1A oligomerizes with GPR39 [48,49] and with galanin receptor type 1 (GalR1) [50]. A trimer consisting of 5-HT1A-GPR39-GalR1 was also demonstrated [48]. When there is lack of GPR39 (in GPR39 KO mice), the trimer 5-HT1A-GPR39-GalR1 and the dimer 5-HT1A-GPR39 cannot exist, while there are 5-HT1A monomers and 5-HT1A-GalR1 dimers [50]. TC-G 1008 may then act via 5-HT1A monomers and/or 5-HT1A-GalR1 dimers. In WT mice, the formation of the trimer may prevail. Importantly, in the 5-HT1A-GPR39-GalR1 trimer, the effects of ZnCl_2_ or the 5-HT1A agonist, 8-OH-DPAT, on serum response element (SRE) were abolished [48]. The presence of trimer in WT mice may inhibit the effects of GPR39 agonist on another transcription factor, i.e., CREB. 

In ZnD mice, there was reduced expression of the GPR39 protein in the hippocampus [12,25]. Like in GPR39 KO mice, TC-G 1008 may then act via 5-HT1A, leading to CREB activation. The increase in pCREB/CREB observed in the present study in the hippocampus of kindled mice fed the ZnD diet and treated with TC-G 1008 might have therefore resulted from compound action at 5-HT1A. A synergistic interaction between increased amount of zinc in the ZnA diet and TC-G 008 may in turn explain a tendency towards increased pCREB/CREB and BDNF levels in the hippocampus of kindled mice fed the ZnA diet that received TC-G 1008 during this chronic paradigm. 

BDNF, the protein product of a gene which is among targets for CREB, plays a protective role in the survival of neurons and stimulates the formation of new neurons, i.e., the process of neurogenesis [51]. Zinc deficiency has profound consequences for decreasing neurogenesis [52]. Chronic ZnD diet decreased the levels of BDNF in the hippocampus of rodents [34,35] but contrasting findings have also been reported [53]. In turn, treatment with zinc increased BDNF in the hippocampus but the effects of zinc administration on increasing BDNF level depend on the dose, mode of administration (acute/chronic), and time of sampling for the analysis [54,55]. Increasing BDNF seems promising in various diseases, but in epilepsy it may promote epileptogenesis [56]. It is plausible that a prolonged ZnA diet coupled to TC-G 1008 administration might have contributed to enhancing epileptogenesis by mechanisms associated with neurogenesis. 

Consistent with the hypothesis of increased BDNF signaling promoting epileptogenesis, we previously found in genetically unmodified (Swiss Albino) mice a trend towards increased p-CREB/CREB and p-TrkB/TrkB ratios in the hippocampus of mice that underwent kindling induced by 19 injections of PTZ (40 mg/kg) (the comparison between VEH kindled and VEH non-kindled mice). Chronic treatment with VPA (150 mg/kg), TC-G 1008 (10 mg/kg), or ZnCl_2_ (8 mg Zn/kg) decreased p-CREB/CREB and p-TrkB/TrkB ratios in the hippocampus of kindled mice (Figure S10, supplemental data) [16]. Here, after 14 injections of PTZ (40 mg/kg) there was only a small tendency towards an increased p-CREB/CREB ratio (in VEH kindled vs. VEH non-kindled mice). In other words, the longer model might have contributed to a trend towards the increased activation of CREB and TrkB. The greater number of PTZ injections as well as different amount of zinc in the diet might have therefore accounted for the differences between our previous (Figure S10, supplemental data) [16] and present study in terms of the effects of drugs administered chronically in Swiss Albino mice during PTZ-kindling on protein expression in the hippocampus. 

Two cross-sectional studies showed a decreased dietary intake of zinc in children with intractable epilepsy compared to healthy children [57,58]. In preclinical studies on seizures/epilepsy, the ZnD diet for rats or mice contained between 0.4 mg and 2.7 mg Zn/kg, while the control diet contained between 34 and 44 mg Zn/kg [59]. Chronic (4–8 weeks) ZnD diet increased susceptibility to seizures induced by KA injection in ddY mice and Wistar rats [60], and by NMDA injection in ddY mice [61], but not to PTZ in ddY mice [62]. Moreover, ZnD diet (2.7 mg Zn/kg vs. 44 mg Zn/kg) decreased the seizure threshold to penicillin and lithium chloride-pilocarpine-SE further decreased the threshold for penicillin in ZnD-mice [12]. These observations indicated that the occurrence of behavioral seizures in the ZnD animals depend on the mechanism of action of the chemoconvulsant. Based on these data, we did not anticipate differences in either seizure threshold or epileptogenesis between mice fed the ZnA and ZnD diet, but we hypothesized that TC-G 1008 would act differently in mice depending on the diet. 

Administration of zinc in preclinical models of seizures/epilepsy exerted either a pro- or anti-seizure effect, depending on the dose and form of zinc, its route of administration, and the applied model [16,59]. In our previous study in which mice were fed the diet of 25 mg Zn/kg, both doses of ZnCl_2_: 8 and 16 mg Zn/kg decreased the seizure threshold in the MEST test [16]. Here, 4 and 16 mg Zn/kg decreased the seizure threshold in ZnD mice, while the dose of 8 mg Zn/kg was ineffective. After 4 weeks of the ZnD diet, the mean body weight of mice in the VEH group was 35.5 g, the mean body weight in the ZnD Zn 8 group was 33.7 g, while it was 31.2 g in the ZnD Zn 16 group. The mean body weight of ZnA mice treated with VEH was 40 g, the mean body weight of ZnA Zn 8 was 39.3 g, while it was 40 g in the ZnA Zn 16 group (data not shown). Weight loss, in addition to decreased serum zinc, is a feature of severe zinc deficiency [63]. These data indicated that the ZnD diet worked in all groups. It is possible that administration of ZnCl_2_ was not efficient in several mouse in the ZnD Zn 8 group, leading to variability within this group and the variability in biological response.

Chronic (a 2-week) treatment with ZnSO_4_ p.o. at doses corresponding to 200 mg Zn/kg decreased the number of kindled animals from 66.7 to 14.3% and reduced the seizure severity score in the PTZ kindling model [64]. Furthermore, the chronic (4-week) continuous infusion of ZnCl_2_ solution delayed the development of behavioral seizures in a kindling model induced by electrical stimulation and inhibited progression of after discharge duration [65]. We found increased mortality during PTZ-kindling in mice receiving ZnCl_2_ in both mice fed the ZnA and ZnD diet, which excludes this form and dose of zinc from being regarded as anti-epileptogenic strategy.

In the brain, the highest levels of zinc are in the hippocampus [66]. Zinc deficiency impairs brain function in humans [67] and several weeks of a ZnD diet produce behavioral alterations in experimental animals [68]. However, the changes in behavior may not be accompanied by changes in total zinc brain concentration. We found decreased total zinc concentration in the hippocampus and cortex after a 4-week dietary zinc restriction [36] but other studies did not [37,38]. Decreased total zinc in the hippocampus was observed by other researchers after a longer period of the ZnD diet administration, i.e., after 12 weeks [37]. On the other hand, in the R6/1 genetic mouse model of Huntington’s disease decreased total zinc in the hippocampus and cortex was observed [69]. However, the mice that displayed decreased zinc in these brain regions at the age of 16 weeks did not show cognitive impairment [69]. These studies suggested that changes in the brain zinc may follow or precede changes in animal’s behavior induced by zinc deficiency.

Using the ICP-MS method we analyzed semi-quantitatively two brains per group of mice that were subjected to the kindling model. The normalized zinc signal in the hippocampus was 0.13 in both mice fed the ZnA and ZnD diet for >8 weeks, while total zinc concentration in serum was decreased by ca. 50% in the ZnD mice. Using the same method, we previously did not observe significant changes in total zinc in the hippocampus of GPR39 KO mice subjected to the PTZ-kindling model of epilepsy. Moreover, administration of TC-G 1008 did not produce hippocampal alterations in total zinc in either kindled GPR39 KO or WT mice [16]. Altogether, these results suggest that the hippocampus is indeed protected from the effects of zinc deficiency [70]. Alternatively, the changes in the brain may only involve other pools of zinc such as free zinc ions [16,59].

## 5. Conclusions

We replicated our previous findings that TC-G 1008 decreased the seizure threshold in the MEST test and facilitated epileptogenesis in the PTZ-kindling model [16]. Replication was necessary as aggravation of either acute seizures or the chronic process of epilepsy development by a compound acting at GPR39 is contrary to the prevailing hypothesis on GPR39 activation being a potential new therapeutic strategy for treating seizures/epilepsy [15]. The ZnD diet abolished the chronic effects of TC-G 1008 on the maximal seizure score and the percentage of fully kindled mice in the PTZ-kindling model of epilepsy, thus showing that dietary zinc is an important modulator of the effects mediated by GPR39. Conversely, the acute effects of TC-G 1008 on the seizure threshold were observed in mice fed the ZnD diet. The involvement of molecular targets other than GPR39 in the mechanism of action of TC-G 1008 in the MEST test, the involvement of a particular signaling pathway downstream of GPR39 or the process of oligomerization between GPCRs might have accounted for differential regulation of the acute/chronic effects of TC-G 1008 in the acute test/chronic model by dietary zinc. Our data showed that the amount of zinc in a diet is the factor contributing to the behavioral effects of TC-G 1008 in vivo. Diet may be a factor determining the in vivo effects of compounds which were shown to be modulated by zinc in vitro. Moreover, dietary zinc is a factor contributing to the biochemical effects of TC-G 1008. Reporting the composition of a diet, including the content of essential metals such as zinc would be a good practice, as it may shed new light on the interpretation of the results of an experimental work.

## Data Availability

The data that support the findings of this study are available from the corresponding author upon reasonable request.
